# Chronic Heat Stress Induces Stage-Specific Molecular and Physiological Responses in Spotted Seabass (*Lateolabrax maculatus*): Focus on Thermosensory Signaling and HPI Axis Activation

**DOI:** 10.3390/biology15020113

**Published:** 2026-01-06

**Authors:** Guozhu Zhang, Hao Niu, Xiangkai Tang, Kaile Wang, Xue Xia, Xiu Fang, Xiaojie Wang

**Affiliations:** 1 National Demonstration Center for Experimental Fisheries Science Education, Shanghai Ocean University, Shanghai 201306, China; 2 Key Laboratory of Exploration and Utilization of Aquatic Genetic Resources, Ministry of Education, Shanghai Ocean University, Shanghai 201306, China; 3 International Research Center for Marine Biosciences, Ministry of Science and Technology, Shanghai Ocean University, Shanghai 201306, China; 4 Fujian Provincial Key Laboratory of Breeding Lateolabrax Japonicus, Fuding 355200, China

**Keywords:** *Lateolabrax maculatus*, high temperature, heat stress, developmental stage, behavioral, thermosensory, HPI axis, appetite

## Abstract

Global warming and the increasing frequency of marine heatwaves (MHWs) threaten marine ecosystems and aquaculture. For the economically important spotted seabass (*L. maculatus*), the neuroendocrine basis of its stage-specific thermal responses has yet to be elucidated. This study examined the transcriptomic, physiological, and behavioral adaptations to chronic heat stress in late larval and late juvenile seabass over 14 days. After thermal acclimation, larvae demonstrated a marked behavioral shift, preferring warmer waters (26–34 °C). While heat stress upregulated key thermosensory genes (e.g., *trpv1*, *trpv4*) in the brain across both stages, it induced distinct expression profiles in the skin, suggesting a developmental transition from peripheral to central dominance in thermosensation. Brain transcriptomics revealed stage-specific pathway activation: juveniles engaged in neuroactive ligand-receptor interactions and MAPK signaling, whereas larvae showed enrichment in phosphatidylinositol signaling and protein processing. Both stages showed activation of the hypothalamic-pituitary-interrenal (HPI) axis (upregulation of *crh*, *crhr1*, *crhr2*, *pomc*) and heat shock response (*hsp70*, *hsp90*), accompanied by elevated serum cortisol. Notably, energy metabolism diverged significantly: larvae maintained appetite and developed hyperglycemia, while juveniles exhibited severe feeding suppression and hypoglycemia, which was correlated with differential regulation of appetite genes (*npy*, *orexin*, *cck*). Our results elucidate the distinct neuroendocrine mechanisms underlying thermal acclimation in *L. maculatus* and provide a scientific basis for developing climate-resilient aquaculture practices for this species.

## Simple Summary

Global warming poses a significant threat to aquaculture. Marine heatwaves (MHWs), in particular, endanger the spotted seabass (*Lateolabrax maculatus*), a commercially important marine species in Northeast Asia. We previously did not understand how its neuroendocrine system copes with heat across different developmental stages. So, we conducted an experiment: we exposed late larvae and late juveniles of this seabass to high temperatures for 14 days and tracked their changes. The two stages (late larvae and juveniles) exhibited distinct thermosensory and behavioral responses to heat stress: larvae relied more on their skin to “feel” temperature, while juveniles depended more on their brains. Both activated stress responses (like higher stress hormones), but larvae kept eating normally (with higher blood sugar), while juveniles ate about 80% less (with lower blood sugar). These findings not only help us better understand how spotted seabass acclimatize to heat as they grow, but also provide practical references for its aquaculture—helping farmers take targeted measures to protect seabass survival and yields when MHWs occur, which has implications as regards coping with climate change’s impacts on aquatic production.

## 1. Introduction

Water temperature is a critical environmental factor governing fish survival, development, and growth [1]. However, excessively high temperatures pose severe threats to fish populations [2]. According to the World Meteorological Organization’s 2024 climate report, global ocean heat content has reached a record high since measurements began in 1958, surpassing the previous record set in 2023. Notably, each of the past eight consecutive years (2017–2024) has set a new record for oceanic heat accumulation. Moreover, the rate of ocean warming more than doubled between 2005 and 2024 compared to the period from 1960 to 2005 [3], highlighting an accelerated pace of marine thermal change. Concurrently, the frequency and intensity of marine heatwaves (MHWs)—defined as prolonged periods of anomalously high sea surface temperatures that severely disrupt marine ecosystems [4]—have increased significantly under ongoing global climate change. MHWs destabilize ecosystems and force fish to shift their habitats to avoid lethal temperatures. This is evidenced by distribution data from the North Sea: over the past 25 years, nearly two-thirds of fish species have shifted northward or to deeper waters, a pattern strongly correlated with rising sea surface temperatures and indicative of climate change as the primary driver [5]. Importantly, this temperature rise also exerts adverse effects on aquaculture productivity—a trend that not only threatens economic output but also undermines regional food security [6].

The link between warming oceans and aquaculture decline is the direct disruption of fish physiology [7]. As poikilothermic organisms, fish rely primarily on external environmental conditions to regulate body temperature. Although fish possess thermoregulatory mechanisms involving the hypothalamus (thermal perception), brainstem (response coordination), and spinal cord (mediating behaviors such as thermal avoidance), the capacity of this neuroendocrine system to maintain internal thermal homeostasis is inherently limited: it can only buffer against short-term and mild temperature fluctuations, not sustained or extreme warming [8]. Consequently, fish are highly sensitive to even subtle shifts in ambient water temperature. Temperatures exceeding this homeostatic threshold trigger a cascade of metabolic and physiological perturbations, including elevated energy expenditure, impaired immune function, and disrupted nutrient absorption. These perturbations often lead to homeostatic breakdown and heightened stress levels [9]. This physiological vulnerability not only drives the observed shifts in species distributions but also directly reduces aquaculture yields [10].

Temperature perception in animals is mainly mediated by thermosensitive ion channels in the sensory nervous system [11]. Transient receptor potential (TRP) channels are key thermal ion channels widely distributed across various organisms, with distinct expression patterns in peripheral sensory organs and central nervous system (CNS) (e.g., brain, hypothalamus) that underpin temperature sensing [12]. Within the TRPV subfamily, TRPV1, the first identified thermosensitive TRP channel, is activated by elevated temperatures [13]. In Atlantic salmon (*Salmo salar*), trpv1 is expressed in both peripheral tissues (skin sensory receptors) and central brain regions (pineal gland, hypothalamus), with its expression modulated by environmental temperature changes [14]. TRPV4 is expressed in sensory neurons or skin keratinocytes, and it responds to warm temperatures and hypotonic pressure by linking peripheral heat signals with CNS signals [15]. In the TRPM subfamily, TRPM2, a calcium-permeable cation channel, is predominantly expressed in the hypothalamus (central thermal integration center) and peripheral immune cells of fish [16]. In Antarctic fishes (Cryonotothenioidea), the temperature-sensing function of TRPM4 has been indicated through selective pressure analysis, with expression detected in both skin sensory ganglia and brainstem neurons, thereby providing molecular evidence for adaptive thermal perception [17]. TREK channels: As temperature-sensitive potassium channels, TREK1 and TREK2 are widely distributed in the peripheral sensory neurons and CNS of fish [18]. Other thermosensitive receptors, such as the ionotropic glutamate receptor GluK2, have also been reported in the peripheral sensory neurons of mice [19], but their distribution in fish remains understudied. At the organismal level, studies on salmonids (e.g., Atlantic salmon, rainbow trout) indicate that chronic thermal exposure alters thermal sensitivity, which reflects a sophisticated capacity to perceive and respond physiologically to environmental temperature changes [20].

In mice, thermal activation of the TRPM2 channel stimulates excitatory neurons to release glutamate into the paraventricular nucleus of the hypothalamus (PVH). There, glutamate acts on corticotropin-releasing hormone (CRH) neurons, inducing depolarization, enhancing Crh gene expression, and thereby promoting CRH release into the pituitary portal system [21]. This CRH release initiates a cascade along the hypothalamic-pituitary-interrenal (HPI) axis. Acting through its receptors (CRHR1 and CRHR2), CRH integrates environmental stress to coordinate adaptive responses [22]. This CRH signal then triggers the pituitary to secrete pro-opiomelanocortin (POMC)-derived peptides and ultimately release cortisol [23]. Cortisol, a key hormone in fish, regulates stress, metabolism, and osmoregulation [24]. It typically induces transient hyperglycemia by stimulating glycogen breakdown and limiting glucose utilization [25]. However, prolonged heat stress disrupts this pattern by impairing metabolism and digestion, suppressing appetite, and increasing maintenance energy costs [26]. The resulting energy deficit can cause hypoglycemia. As shown in rainbow trout (*Oncorhynchus mykiss*), chronic heat stress suppresses feeding and growth, underscoring its threat to aquaculture [27].

Fish experience substantial fluctuations in environmental temperature, and heat shock proteins (HSPs) play a crucial role in helping them cope with heat stress and other environmental challenges [28]. HSPs are a group of evolutionarily conserved molecular chaperones, with HSP70 and HSP90 being the most conserved and functionally significant families [29]. Studies in doctor fish (*Garra rufa*) have also shown that HSPs protect cells from stress-induced damage by facilitating protein folding, repairing misfolded proteins, and preventing protein aggregation under elevated temperature conditions [30]. Therefore, HSPs are widely recognized as reliable biomarkers for assessing organismal stress responses, with intracellular accumulation levels as part of stress intensity [31]. Climate change leads to an increase in high temperature. This environmental factor that can change fish appetite and metabolism, affecting fish population in both wild and aquaculture facilities [32].

The spotted seabass (*L*. *maculatus*) is a commercially important marine species native to Northeast Asia, widely cultivated in southern China for its rapid growth and adaptability. According to the China Fishery Statistical Yearbook 2024, its annual production reached 246,900 tons in 2023, reflecting a consistent upward trend. This species thrives within an optimal temperature range of 16–27 °C [33]. However, its habitat is increasingly threatened by coastal MHWs and rising sea surface temperatures, which have been recorded as high as 31–32 °C in recent years [34,35]. While existing studies on spotted seabass have primarily documented general physiological and biochemical responses to heat stress [36,37,38], a critical knowledge gap remains regarding the underlying neuroendocrine mechanisms—particularly how these responses differ across developmental stages.

Thermal tolerance is known to vary significantly with developmental stages, a pattern observed in species like Atlantic herring (*Clupea harengus*) and European sea bass (*Dicentrarchus labrax*), where high temperature exerts differential impacts on the neuroendocrine system [39,40,41]. The HPI axis, central to the stress response, is structurally formed but functionally immature in larvae, whereas it attains full stress-responsive capacity in late juveniles [42]. This disparity in HPI axis maturity is a key factor underlies stage-specific differences in stress-linked physiological outcomes, such as appetite regulation [43]. Therefore, this study focused on two critical stages—late larvae and late juveniles—to elucidate the stage-specific mechanisms of thermosensation and neuroendocrine regulation in spotted seabass under chronic heat stress. The findings are expected to provide critical insights for developing adaptive aquaculture strategies for *L. maculatus* in a warming climate.

## 2. Materials and Methods

### 2.1. Fish and Experimental Design

Two independent experiments were conducted using late larval and juvenile stages of spotted seabass. In experiment 1, ninety healthy late larvae (0–1 month post-hatch; mean body weight (BW): 2.2 ± 0.3 g; mean total length (TL): 4.0 ± 0.1 cm) were randomly distributed among six 300 L tanks, which were equally allocated to two temperature-controlled systems (three tanks per system). Within each system, tanks were assigned as either control or experimental groups (three tanks per group in total). In experiment 2, fifty-four healthy late juveniles (11–12 months post-hatch; BW: 393.1 ± 30.5 g; TL: 34.7 ± 2.8 cm) were utilized, similarly distributed into another set of six 1000 L tanks assigned to separate temperature-controlled systems. Again, three tanks served as the control group and three as the experimental group. For both experiments, all subsequent rearing management, sampling time points, and experimental procedures were identical between the control and experimental groups, with the only difference being the temperature conditions applied. This design ensured that comparisons between groups were based solely on the manipulated variable.

### 2.2. Acclimation and Rearing Conditions

Prior to the experiment, all fish were acclimated for 3 days (d) under consistent water quality conditions (dissolved oxygen > 6 mg/L; pH 7.8 ± 0.1; salinity: 31.0 ± 0.2). Heat stress was induced by gradually increasing water temperature from 24.0 ± 0.5 °C to 33.1 ± 0.5 °C for larvae, and from 20.1 ± 0.5 °C to 30.2 ± 0.5 °C for juveniles, at a rate of 1 °C per 12 h. Following acclimation, each group was maintained at its target temperature for 14 d. Fish were fed twice daily (06:00 and 17:00) to satiation with Tongwei Carnivorous Fish Compound Feed (Model 8901; crude protein ≥ 48%, crude fat ≥ 6%; Tongwei Co., Ltd., Chengdu, China), using pellet sizes appropriate for each life stage (1.0 mm for larvae, and 7.0 mm for juvenile). For larvae, unconsumed feed was collected and counted 30 min after each of the two daily feedings, whereas for juveniles, uneaten pellets were collected each morning to calculate daily feed intake. To maintain water quality, 30% of the tank volume was replaced daily with temperature-equilibrated, aerated seawater. Feeding was suspended 24 h before final sampling to minimize stress in fish.

### 2.3. Behavioral Tests

Behavioral tests were conducted both before and after the 14-day thermal exposure. At each time point, 45 larval fish were randomly selected and divided into three groups (n = 15 per group). Prior to the heating experiment, two behavioral assays were performed: one using a constant-temperature tank (24 °C) and another using a thermal gradient tank. Following the 14-day thermal exposure, only the thermal gradient assay was repeated.

A rectangular tank (120 × 20 × 20 cm) was used to establish the thermal gradient. The tank was divided into six interconnected chambers by transparent acrylic partitions, each featuring a central circular opening (5 cm diameter) 10 cm above the bottom to allow for fish movement. Before testing, fish were acclimated in the apparatus for 2 h under a uniform temperature (24 °C) to reduce stress. In the constant-temperature assay, the entire tank was maintained at 24 °C. In the thermal gradient assays (post-acclimation), a stable gradient from approximately 14.0 ± 0.8 °C (Chamber 1) to 34.0 ± 1.7 °C (Chamber 6) was established. During each 8 h experiment, water temperature in each chamber and fish distribution were recorded at 30 min intervals (16 time-points total) using calibrated digital sensors and visual assessment, respectively, without disturbing the animals.

### 2.4. Sample Collection

Fish were anesthetized with MS-222 (tricaine methanesulfonate; Sigma-Aldrich, St. Louis, MO, USA) using concentration ranges adjusted to body size: 80–120 mg/L for larvae and 120–160 mg/L for juveniles. Following anesthesia, blood was drawn from the caudal vein with a disposable syringe for serum cortisol and glucose analysis. Each fish was then dissected to collect the whole brain and skin samples.

### 2.5. RNA Extraction and RT-qPCR

Total RNA was extracted from the brain, and skin tissues using TRIzol reagent (Sangon Biotech, Shanghai, China, Order NO. B610409) following the manufacturer’s instructions. RNA concentration and purity were assessed on a NanoDrop One spectrophotometer (Thermo Fisher Scientific, Waltham, MA, USA) via A260/A280 and A260/A230 ratios, and integrity was verified by agarose gel electrophoresis. cDNA synthesis was conducted following the instructions provided with the HiScript III SuperMix for qPCR (+gDNA wiper) kit (Vazyme, Nanjing, China, Cat. No. R323). The synthesized cDNA was stored at −20 °C until use. Quantitative real-time PCR (qPCR) was performed in triplicate using SYBR Green Master Mix (Vazyme, Nanjing, China) on a CFX96 System (Bio-Rad, Hercules, CA, USA) with gene-specific primers (Table 1). The 2^−ΔΔCt^ method was used to calculate relative gene expression, normalized to *β-actin*, which has been reported as a stable reference gene in spotted seabass [44] and whose stability was further verified in our study. The primers, presented in Table 1, were designed using Primer-BLAST, and the specificity of amplification was confirmed by a single peak in the melt curve, and no non-specific amplification was detected.

### 2.6. RNA Sequencing and Analysis

RNA-seq libraries were prepared from total brain RNA in two stages. Briefly, mRNA was enriched using poly-T oligo-attached magnetic beads and fragmented. First- and second-strand cDNA were synthesized using random hexamers and DNA Polymerase I, respectively. The resulting cDNA underwent end repair, adenylation, and adapter ligation. Libraries were size-selected (370–420 bp) using AMPure XP beads (Beckman Coulter, Brea, CA, USA), PCR-amplified with index primers, and quality-checked on an Agilent Bioanalyzer 2100 (Agilent Technologies, Santa Clara, CA, USA). Raw sequencing reads were quality-controlled with fastp (v0.23.2, developed by Hua Chen’s group at Peking University Health Science Center) to remove adapters, poly-N sequences, and low-quality bases (Q < 20), yielding high-quality clean reads. RNA-seq analysis was performed on the NovoCloud platform (Novogene Co., Ltd., Beijing, China). Raw sequencing data underwent quality control using the platform’s built-in pipeline. High-quality reads were aligned to the spotted seabass reference genome (GigaDB: 100458) using HISAT2 (version 2.2.1). Gene counting was carried out with the platform’s featureCounts software (version 2.0.3) based on the genome annotation file (GFF3 format). Gene expression levels were normalized using Z-score transformation to enable cross-sample comparison and visualization. Functional annotation of genes was directly obtained from the reference genome-associated annotations and supplemented by aligning sequences to public databases (Nr, Swiss-Prot, KEGG, and GO) using the integrated BLAST tools on the platform. Differentially expressed genes (DEGs) were identified using DESeq2 (version 1.40.2) with thresholds of |log_2_(fold change)| ≥ 1 and adjusted *p*-value (Padj) < 0.05. The results were visualized via principal component analysis (PCA), volcano plots, and heatmaps for validation.

### 2.7. Serum Cortisol and Glucose Analysis

Serum cortisol and glucose levels were quantified using commercial assay kits (cortisol: H094-1-1; glucose: A154-1-1; Jiancheng Bioengineering Institute, Nanjing, China) according to the manufacturer’s protocols. Absorbance was measured using a SpectraMax iD3 Multi-Mode Microplate Reader (Molecular Devices, San Jose, CA, USA), and analyte concentrations were determined from standard curves analyzed in parallel. All samples were run in duplicate.

### 2.8. Statistical Analysis

Statistical analyses were performed using IBM SPSS Statistics (version 25.0). The core analysis of this study involved two-group comparisons for each life stage (control group vs. treatment group). All datasets were tested for normality using the Shapiro–Wilk test. For comparisons between two independent groups, when the data did not follow a normal distribution, a Mann–Whitney test was used. All tests were considered significant at *p* < 0.05. Data are presented as mean ± SEM. Graphs were generated using GraphPad Prism software (version 8.4.3).

## 3. Results

### 3.1. Shift in Thermal Preference Following Acclimation in Late Larvae

**Figure 1:**
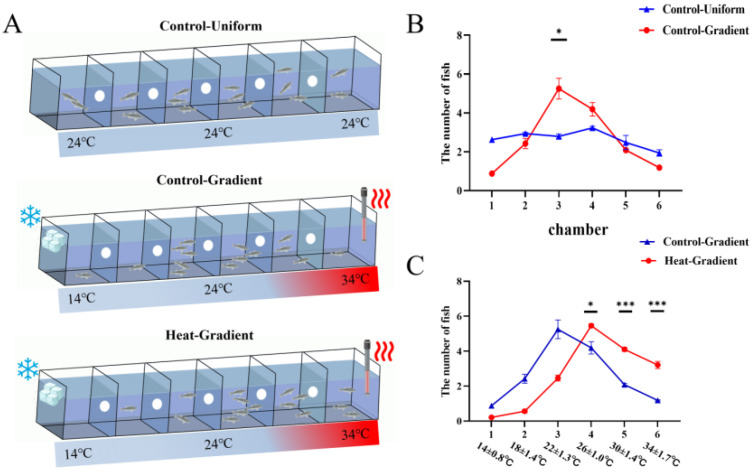
A shift in thermal preference of late-stage spotted seabass larvae following heat acclimation. (**A**) Schematic of the experimental setup and representative distributions of larvae for the three key conditions: uniform temperature (24 °C), thermal gradient (control), and thermal gradient post-heat acclimation (n = 15). (**B**,**C**) Quantitative distribution profiles of larvae within the thermal gradient. (**B**) Control group under gradient versus uniform temperature conditions. (**C**) Comparing of control and heat-acclimated groups under gradient conditions. Data represent the mean percentage of individuals per chamber (n = 15). (* *p* < 0.05, *** *p* < 0.001).

Having established that late larvae exhibit a random distribution under uniform temperature conditions (Figure 1A), we next examined their behavior in a thermal gradient (14–34 °C). Under gradient conditions, a significant majority (88.06%) of larvae selected the 22–26 °C range (*p* < 0.05; Figure 1B), defining their baseline thermal preference. Subsequently, we investigated the effect of heat acclimation and observed a significant redistribution: following 14 days of acclimation, 71.32% of the larval population shifted to occupy the warmer 26–34 °C range (*p* < 0.05; Figure 1C). This result demonstrates that prior thermal experience significantly modulates preferential temperature selection in late-stage larvae.

### 3.2. Distinct Transcriptomic Responses to Heat Stress in the Brain Across Developmental Stages

**Figure 2:**
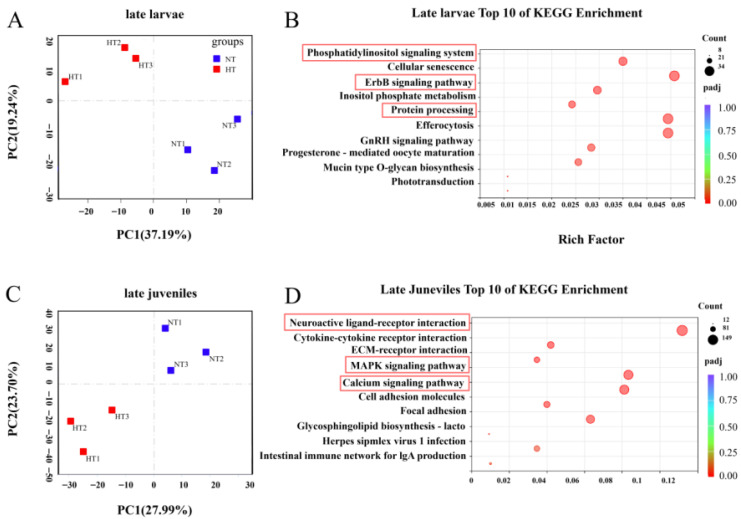
Developmental stage-specific transcriptomic responses to heat stress in the spotted seabass brain. (**A**,**C**) Principal component analysis (PCA) of brain transcriptomes from late larvae (**A**) and late juveniles (**C**) under control and heat-stress conditions. (**B**,**D**) KEGG pathway enrichment analysis showing the top 10 significantly enriched pathways in the brain of late larvae (**B**) and late juveniles (**D**) after heat stress.

We observed distinct transcriptomic responses to heat stress in the brain across developmental stages. PCA indicated significant differences in global transcriptome profiles before and after heat stress in both late larvae (Figure 2A) and late juveniles (Figure 2C). Stage-specific responses were evident from KEGG pathway enrichment analysis. In late larvae, the most significant enriched pathways were the phosphatidylinositol signaling system, ErbB signaling pathway, and protein processing (Figure 2B). In late juveniles, however, the response involved multiple key signaling and immune-related pathways, with the most prominent being neuroactive ligand-receptor interaction, the MAPK signaling pathway, and the calcium signaling pathway (Figure 2D).

### 3.3. Expression of Genes Related to Temperature Sensing

**Figure 3:**
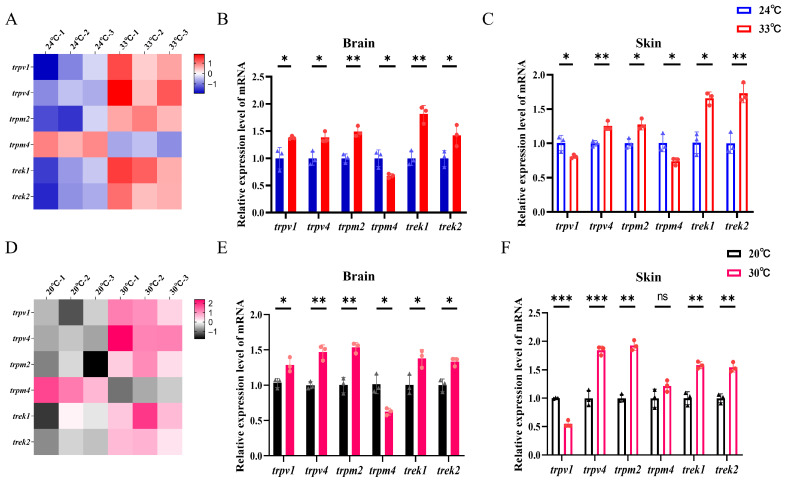
Expression of thermosensory genes in spotted seabass under control and heat-stress conditions. (**A**–**C**) Late larval stage: (**A**) Heatmap of RNA-seq profiles for thermosensory gene expression in the brain. (**B**,**C**) Relative mRNA expression levels of thermosensory genes in the brain (**B**) and skin (**C**). (**D**–**F**) Late juvenile stage: (**D**) Heatmap of RNA-seq profiles for thermosensory gene expression in the brain. (**E**,**F**) Relative mRNA expression levels of thermosensory genes in the brain (**E**) and skin (**F**). (n = 3; * *p* < 0.05, ** *p* < 0.01, *** *p* < 0.001; ns, not significant, *p* > 0.05).

After 14 days of heat stress, transcriptomic profiling of late larvae brain tissue revealed significant alterations in the expression of thermosensory-related genes, including members of the TRP channel family (*trpv1*, *trpv4*, *trpm2*, *trpm4*) and TREK potassium channels (*trek1*, *trek2*; Figure 3A). Compared with the room-temperature controls, heat-treated larvae exhibited significant upregulation of *trpv1*, *trpv4*, *trpm2*, *trek1*, and *trek2* mRNA levels in the brain (all *p* < 0.05), while *trpm4* expression was markedly downregulated (*p* < 0.05; Figure 3B). A comparable expression pattern was observed in larval skin tissue, where heat stress led to significant upregulation of *trpv4*, *trpm2*, *trek1* and *trek2* (*p* < 0.05), but significant downregulation of *trpv1*, *trpm4* (Figure 3C).

In late juveniles subjected to the same heat treatment, brain transcriptome profiling also indicated substantial alterations in thermosensitive gene expression (Figure 3D). Consistent with the larval response, the heat-stressed juveniles exhibited upregulated mRNA levels for *trpv1*, *trpv4*, *trpm2*, *trek1*, and *trek2* (all *p* < 0.05), alongside downregulated trpm4 expression (*p* < 0.05; Figure 3E). In skin tissue, however, the expression profile differed: thermal stress significantly elevated *trpv4*, *trpm2*, *trek1* and *trek2* transcript levels (all *p* < 0.05), suppressed *trpv1* expression (*p* < 0.05), but did not significantly affect *trpm4* (Figure 3F).

### 3.4. Stress Pathway Activation and Systemic Physiology Following Heat Stress

**Figure 4:**
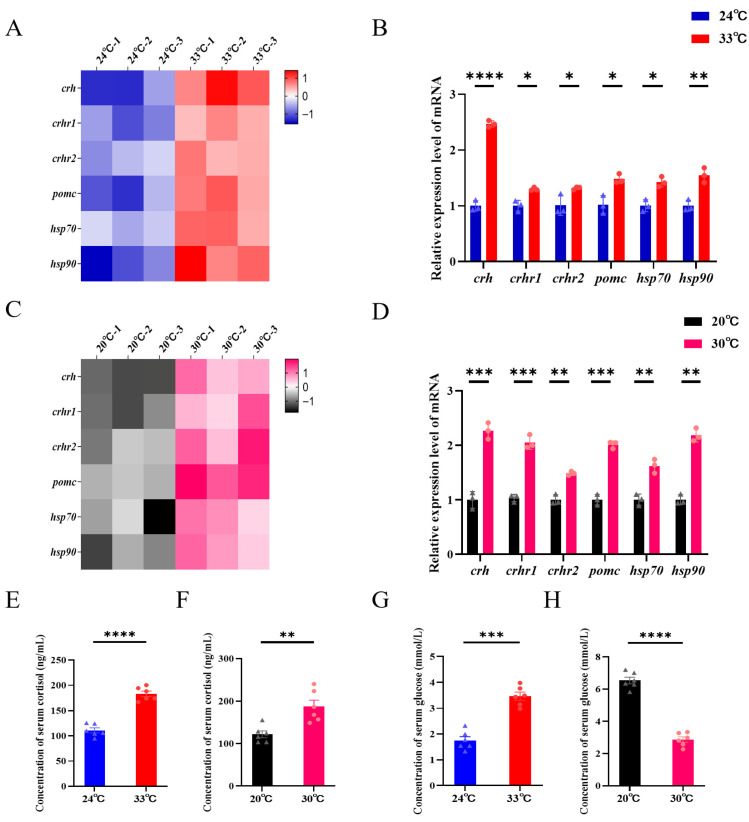
Neurological and systemic stress responses to heat stress in spotted seabass. (**A**,**B**) Late larval brain: (**A**) RNA-seq heatmap and (**B**) relative mRNA expression levels of HPI axis-related and HSP genes under control and heat stress conditions (n = 3). (**C**,**D**) Late juvenile brain: (**C**) RNA-seq heatmap and (**D**) relative mRNA expression levels of the same gene set (n = 3). (**E**–**G**) Serum cortisol levels in late larvae (**E**) and late juveniles (**F**) (n = 6). (**F**–**H**) Serum glucose levels in the late larvae (**G**) and late juveniles (**H**) (n = 6). (* *p* < 0.05, ** *p* < 0.01, *** *p* < 0.001, **** *p* < 0.0001).

In late larvae, 14 days of heat stress significantly upregulated brain genes associated with CNS signaling and stress adaptation, as determined by transcriptomic profiling. Key regulators of neuroendocrine responses (e.g., *crh*, *crhr1*, *crhr2*, *pomc*) and cellular stress defenses (e.g., *hsp90*, *hsp70*) were significantly upregulated compared to the control group (Figure 4A,B; *p* < 0.05).

A comparable transcriptional response was observed in late juveniles, with significant upregulation of the same suite of CNS and cellular stress-related genes (Figure 4C,D; *p* < 0.05), indicating a conserved neuroendocrine stress activation across developmental stages.

At the systemic physiology level, serum cortisol concentration increased significantly by 79.18 ng/mL compared to the control group in heat-stressed late larvae (Figure 4E), and a similar elevation (65.38 ng/mL) was noted in late juveniles (Figure 4F). In contrast, serum glucose levels showed a stage-specific response: heat stress increased glucose by 1.71 mmol/L in late larvae (Figure 4G), but significantly decreased by 3.66 mmol/L in late juveniles (Figure 4H).

### 3.5. Analysis of Feeding Behavior and Brain Appetite-Regulating Gene Expression in Response to Heat Stress Across Developmental Stages

**Figure 5:**
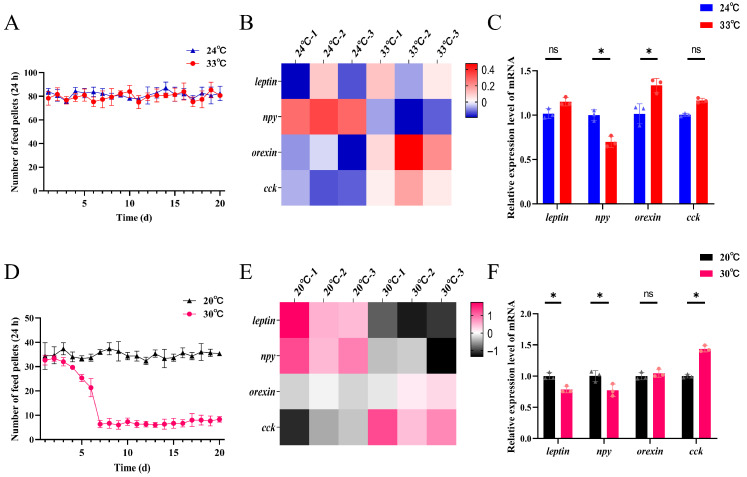
Feeding behavior and appetite-related gene expression in spotted seabass under control and heat-stress conditions. (**A**–**C**) Late larval stage: (**A**) Daily food intake under control and heat stress conditions (n = 12); (**B**) Heatmap and (**C**) relative mRNA expression levels of key appetite-regulating genes in the brain (n = 3). (**D**–**F**) Late juvenile stage: (**D**) Daily food intake under control and heat stress conditions (n = 15); (**E**) Heatmap and (**F**) relative mRNA expression levels of appetite-regulating genes in the brain (n = 3). (* *p* < 0.05. ns, not significant, *p* > 0.05).

Figure 5A indicates no significant alteration in food intake among late larvae under heat stress. However, transcriptomic analysis of the larval brain tissue after 14 days of heat stress revealed notable changes in the expression of several key appetite-regulating genes (Figure 5B). Specifically, while *leptin* and *cck* expression levels did not differ significantly from those of the control group (*p* > 0.05), *npy* was significantly downregulated (*p* < 0.05) and *orexin* was significantly upregulated (*p* < 0.05).

In contrast, a marked reduction in feeding behavior was observed in late juveniles under heat-stress conditions (Figure 5D), with food consumption dropping to approximately one-fifth of that recorded in the control group. Corresponding transcriptomic analysis of juvenile brain tissue following 14 days of heat stress demonstrated significant alterations in the expression of appetite-regulated genes, including *leptin*, *npy*, *orexin*, and *cck* (Figure 5E). Compared with controls, *leptin* and *npy* were significantly downregulated (*p* < 0.05), *cck* was significantly upregulated (*p* < 0.05), while *orexin* expression remained unchanged (*p* > 0.05).

## 4. Discussion

Compared with the control group maintained at normal temperature during the same period, juvenile and adult fish subjected to heat stress showed significant differences at multiple levels. This comparison allows us to clearly attribute the physiological and molecular changes discussed later to high temperature stress, rather than simply individual developmental processes. To identify key genes involved in the ontogenetic shift in thermal perception in spotted seabass, we first employed transcriptome sequencing on brain and skin tissues of late larvae and late juveniles under 14-day heat stress. This approach revealed differential expression of several thermosensitive ion channel genes (e.g., TRP family, TREK channels), which are established as primary molecular mediators of temperature perception [45]. We subsequently focused our analysis on validating and characterizing the expression patterns of these candidate genes.

Our results demonstrate that heat stress significantly modulated the expression of thermosensitive ion channel in a tissue- and developmental stage-specific manner. In the brain, a conserved response was observed, with significant upregulation of *trpv1*, *trpv4*, *trpm2*, *trek1*, and *trek2* in both life stages. The upregulation of heat-sensitive receptors (e.g., TRPV1) in sensory cells lowers their thermal activation threshold, thereby amplifying the perception of heat stimuli. As the central integrator of environmental signals, the brain may further enhance this process by upregulating such receptors, effectively sensitizing the neural circuit to thermal threats. This heightened signal consequently drives stronger activation of downstream neuroendocrine stress pathways, which can ultimately modulate systemic physiological states, including energy metabolism and overall homeostasis [46], while TRPV4, which responds to warm temperatures, could establish a foundational warm-sensing capacity [47]. Furthermore, the marked upregulation of TRPM2, a known hypothalamic heat sensor [48], is predicted to enhance central thermal sensitivity, facilitating the detection of subtle temperature fluctuations. TREK1 and TREK2, which are temperature-sensitive background potassium ion channels, also exhibit co-upregulation in this context. Their activity increases with rising temperature, and the opening of these channels leads to the outflow of potassium ions. This process causes hyperpolarization of the cell membrane, thereby reducing the excitability of neurons and playing an inherent “inhibitory” or “calming” role [49]. The co-upregulation of thermosensitive potassium channels TREK1/2, known modulators of neuronal excitability [50], may concurrently serve to fine-tune neural activity and prevent overexcitation under prolonged heat.

In contrast, a distinct downregulatory pattern was observed for *trpv1* in juvenile skin and *trpm4* in both tissues and stages. Unlike the brain, the skin serves as the primary interface with the thermal environment. Keratinocytes in the skin themselves can sense changes in environmental temperature and can actively participate in temperature sensation and signaling [51]. The downregulation of cutaneous *trpv1* may represent a “desensitization” mechanism to prevent cellular damage, inflammation, or excessive energy expenditure from chronic receptor overactivation [52]. Our research further revealed that during long-term heat acclimation, the expression of genes associated with acute heat pain, such as *trpv1*, undergoes changes. This indicates that long-term temperature adaptation in fish not only involves classical thermoregulatory pathways, like those mediated by HSP and metabolic recombination, but may also reshape the fish’s perception threshold to temperature stimuli. Similarly, TRPM4 channel is a cation channel sensitive to intracellular calcium ions, and its opening leads to sodium ion influx and depolarization of the cell membrane, thereby enhancing neuronal excitability; conversely, the downregulation of *trpm4* in the brain may function to mitigate heat-induced neuronal hyperexcitability, thereby stabilizing neural network activity. In the skin, reduced *trpm4* expression could act as a peripheral desensitization mechanism, attenuating the sustained relay of thermal signals to the CNS and alleviating systemic stress during sublethal heat exposure [53]. A key finding was the pronounced stage-specific expression in the skin, where larvae exhibited significantly higher transcript levels of most thermosensitive channels than juveniles. This suggests a developmental shift in thermal perception strategy: larval seabass appear to rely more heavily on peripheral (skin) sensing, possibly due to a less mature CNS with limited integrative capacity. In contrast, juvenile increasingly depend on central (brain) integration of thermal cues.

Transcriptomic analysis revealed distinct enriched pathways in the brains of late larval and juvenile spotted seabass under heat stress, indicating fundamentally different coping strategies aligned with developmental stage. While larvae prioritized pathways supporting cellular development and proteostasis, juveniles activated processes related to neural integration and systemic stress resistance. In late larvae, the most significantly enriched pathways included phosphatidylinositol signaling, ErbB signaling, and protein processing. The phosphatidylinositol pathway regulates intracellular calcium levels and vesicle transport, which are essential for larval development (e.g., neural differentiation) and stress-induced ion homeostasis [54]. This pathway is central for cells to sense external stimuli (e.g., temperature changes) and convert them into internal signals, affecting downstream events like calcium release and protein kinase C activation via inositol phospholipid metabolism. Under heat stress, its activation may drive large-scale reprogramming of cell signaling in juvenile fish cells, enabling rapid adjustments in energy allocation, cytoskeleton remodeling, and survival signals, which underlies their high developmental plasticity and reliance on rapid physiological adaptation to high temperatures [55]. The ErbB pathway, involved in cell proliferation and differentiation [56], may support larval growth despite heat stress, as larval stages have higher anabolic demands than juveniles. Protein processing is critical for correcting heat-induced misfolded proteins—a function particularly important for larvae, whose immature protein quality control systems are more vulnerable to thermal disruption. Fish will exhibit stage-specific thermal tolerance based on their metabolic and developmental priorities [57]. Its enrichment strongly suggests that the impact of high temperature stress on juvenile fish deeply affects the central nervous system. This is not just a simple stress response, as it may also involve extensive reshaping of the neuroendocrine regulatory network to integrate and coordinate multiple physiological outputs such as feeding behavior, energy metabolism, and stress hormone release, thereby forming systematic behavioral and physiological adaptation strategies. In contrast, in late juveniles, the top enriched pathways included neuroactive ligand-receptor interaction, MAPK signaling, and calcium signaling—pathways critical for neurotransmission, stress signal transduction, and intracellular homeostasis. Neuroactive ligand-receptor interaction, for example, mediates the communication between sensory neurons (e.g., temperature-sensitive cells) and the hypothalamus, enabling juveniles to integrate environmental thermal cues into physiological responses [58]. The MAPK pathway, a key regulator of cell survival and stress resistance, is activated in fish under heat stress to mitigate oxidative damage and protein misfolding [59]. For spotted seabass, late juveniles prioritize neural signal integration and stress resistance (via MAPK and calcium pathways), while late larvae focus on cell development and protein homeostasis (via ErbB and processing pathways). This distinction highlights the need for stage-specific management strategies in aquaculture—for example, larvae require conditions that support protein folding (e.g., stable dissolved oxygen levels to reduce endoplasmic reticulum stress), while juveniles may benefit from supplements that enhance neural stress resistance (e.g., antioxidants).

Our findings demonstrate that heat stress significantly activated the HPI axis, inducing the upregulation of key genes (*crh*, *crhr1*, *crhr2*, *pomc*) and serum cortisol in both stages. This indicates a conserved transcriptional activation of the stress response machinery across developmental stages. However, a striking stage-specific divergence emerged in physiological outcomes: appetite remained unaffected in larvae but was significantly suppressed in juveniles. We propose that this stems from the differential sensitivity of appetite-regulating circuits to CRH signaling. In larvae, the upregulation of *crhr1* was markedly lower than in juveniles, likely resulting in a weaker anorexigenic signal. This, combined with a potent orexigenic signal from upregulated orexin, effectively counteracted the downregulation of *npy*, allowing larvae to maintain energy intake. In contrast, juveniles exhibited a robust *crhr1* response. This potentiated the suppression of feeding by concurrently upregulating the satiety signal *cck* and downregulating the key orexigenic hormone *npy*. The model is supported by the established roles of these molecules: CRH receptors (CRHR1/2) mediate HPI axis activation, and the release of POMC-derived peptides, which centrally regulate energy homeostasis and feeding behavior [60]. Notably, the anorexigenic effects of CRH are primarily driven by CRHR1 activation [61]. The stress response incurred distinct physiological costs, reflected by stage-specific glucose dynamics. Juveniles developed hypoglycemia despite elevated cortisol, a gluconeogenic hormone. High temperatures may force juvenile fish to mobilize more energy for basic maintenance and stress response, resulting in a systemic glucose consumption rate that exceeds the supply capacity of liver gluconeogenesis, and it may induce mild uncoupling or functional impairment of mitochondria, leading to a decrease in glucose oxidation efficiency [62]. The body needs to break down more glucose to meet the same ATP demand, thereby accelerating the consumption of blood sugar. Thus, juveniles prioritize energy for survival (repair) over anabolism. In contrast, larvae maintained feeding, enabling hyperglycemia by fueling both gluconeogenesis and HSP synthesis. While the coordinated changes in appetite-regulatory neuropeptide mRNAs provide compelling transcriptional evidence for altered central appetite signaling under thermal acclimatization, this study is limited to the transcriptomic level. Future studies incorporating measurements of circulating hormone levels and quantification of corresponding neuropeptide proteins will be crucial to directly validate the physiological impact of these expression changes and to establish the functional hierarchy within this endocrine axis.

Behavioral thermoregulation via habitat selection represents a key rapid-response strategy for fish facing thermal variability [63]. In spotted seabass larvae, a clear ontogenetic shift in thermal preference was observed following chronic heat exposure. Initially aggregating within their optimal range of 22–26 °C, the majority of larvae shifted preference toward warmer waters (26–34 °C) after 14 days of acclimation. This behavioral shift is consistent with patterns of thermal sensitivity attenuation observed in other species such as salmon, where prolonged warming diminishes physiological responsiveness to heat. We propose that the larvae’s revised thermal preference stems from an underlying sensory acclimation, which recalibrates their perception of temperature itself. This functional adaptation renders previously stressful temperatures tolerable, reflecting a form of physiological plasticity rather than mere behavioral choice. As a eurythermal coastal species, spotted seabass larvae likely benefit from this form of thermal habituation, which permits exploitation of warmer microhabitats without proportional increases in physiological stress. Ecologically, such plasticity may improve resilience under marine heatwave conditions by broadening thermal niche breadth. In aquaculture contexts, it further suggests an innate capacity for self-adjustment, potentially reducing reliance on active cooling systems during moderate warming periods.

## 5. Conclusions

This study reveals a hierarchical and ontogenetically dependent acclimation pathway in spotted seabass under chronic heat stress, integrating molecular, physiological, and behavioral responses across developmental stages. The acclimation process begins with stage-specific modulation of thermosensitive ion channels (e.g., TRPs, TREKs), which sharpens central thermal perception while inducing peripheral desensitization. Transcriptomic analysis further demonstrated that this sensory input activates distinct systemic programs: larvae prioritized pathways supporting cellular development and proteostasis, whereas juveniles activated processes related to neural integration and stress resistance. This fundamental divergence in strategic priorities underpins the subsequent physiological response. Larvae maintain energy intake and develop hyperglycemia, directing resources toward growth even under stress. In contrast, juveniles suppress feeding and allocate energy to cellular stress responses—including HSP synthesis—resulting in hypoglycemia, reflecting a clear trade-off that favors survival over growth. These divergent physiological strategies culminate in distinct behavioral outcomes in larvae, which shift their thermal preference toward warmer waters after acclimation—an adaptation supported by their sustained energy status and likely sensory recalibration. While behavioral data were not collected for juveniles, their stress-resistant physiological and transcriptomic profile suggests a reduced capacity for similar behavioral flexibility.

Our findings highlight that thermal acclimation is a coordinated whole-organism process shaped by developmental priorities. The distinct transcriptomic signatures confirm that stage-specific metabolic and developmental demands fundamentally dictate the acclimation trajectory. Accounting for these stage-specific pathways is critical for predicting species responses to climate change and developing effective aquaculture management strategies.

## Figures and Tables

**Table 1:** Primer sequence.

Primer.	Forward (5′–3′)	Reverse (5′–3′)
*trpm2*	CGCCTGGTCCAAACTGATCT	AACAGCACGTAGGCAAACAG
*trpm4*	ACCCGTCACCGCATTTTTAG	TGCAACGCCGTCTGACCTTTG
*trpv1*	CGTCGTCCTTGACATCGCTGAG	GCGATCTCTCCTCAAGCCTC
*trpv4*	GGGTGGATGAGGTGAACTGG	GTCTCCGAAGCCGATTGTGGTG
*trek1*	CCTGCCAGCCGTCATCTTCAAG	CCTTGCGTACTGTGACAGGT
*trek2*	GACGGGCGAGTGTATGCATA	TCATTTCCCGAAGAGCTCCATC
*crh*	ATGAAGCTCAATTTACTTGGCACC	TAGTGGAGGGGCAGGTAGTC
*crhr1*	TCTGAGGAGCAGCCAGAGAT	AGCTCGGGGACTTAAACTGC
*crhr2*	TACTCAGGGCAGGGTCTCTC	AAGAGAGAGGGGAGGCAGAG
*pomc*	GAGTGTATCCGGCTCTGTCG	TCTTTAGTCGCCTGTCGCTG
*hsp70*	GACGGAGGGAAGCCCAAAAT	TGGTTTTCCTTCATGCGGGT
*hsp90*	TGGGCATCCATGAGGACTCTT	TCAGCAAGTCTCAAGATGATCC
*leptin*	ATGGACTACACTCTGGCCATC	GGATATCTTCGTGGCGGTACTCTC
*npy*	ATCATGGCGTTCACCTGGACTG	CGGCCTTTCAGACCCTCTTT
*orexin*	TGCTTCGCAAAGTGCTCAAC	GCTGAGGAGGATGCAGACTC
*cck*	CCGAAATCCATCCACCCCAA	TTGGCTTTGGGGTTCAGG
*β-actin*	CAACTGGGATGACATGGAGAAG	AACAGCACGTAGGCAAACAG

## Data Availability

Due to privacy and ethical restrictions, the data supporting the findings of this study are not publicly available. However, upon reasonable request and subject to the approval of the relevant ethical committee, access to the data may be granted. Interested parties can contact the corresponding author for more information regarding data access procedures.
